# Genetic parameters, prediction, and selection in a white Guinea yam early‐generation breeding population using pedigree information

**DOI:** 10.1002/csc2.20382

**Published:** 2020-12-22

**Authors:** Asrat Asfaw, Dotun Samuel Aderonmu, Kwabena Darkwa, David De Koeyer, Paterne Agre, Ayodeji Abe, Bunmi Olasanmi, Patrick Adebola, Robert Asiedu

**Affiliations:** ^1^ International Institute of Tropical Agriculture (IITA) Ibadan Nigeria; ^2^ International Potato Center (CIP) Abuja Nigeria; ^3^ Dep. of Agronomy Univ. of Ibadan Ibadan Nigeria; ^4^ Pan African Univ., Institute of Life and Earth Sciences Univ. of Ibadan Ibadan Nigeria; ^5^ Agriculture and Agri‐Food Canada 850 Lincoln Road, PO Box 20280 Fredericton NB E3B4Z7 Canada

## Abstract

Better understanding of the genetic control of traits in breeding populations is crucial for the selection of superior varieties and parents. This study aimed to assess genetic parameters and breeding values for six essential traits in a white Guinea yam (*Dioscorea rotundata* Poir.) breeding population. For this, pedigree‐based best linear unbiased prediction (P‐BLUP) was used. The results revealed significant nonadditive genetic variances and medium to high (.45–.79) broad‐sense heritability estimates for the traits studied. The pattern of associations among the genetic values of the traits suggests that selection based on a multiple‐trait selection index has potential for identifying superior breeding lines. Parental breeding values predicted using progeny performance identified 13 clones with high genetic potential for simultaneous improvement of the measured traits in the yam breeding program. Subsets of progeny were identified for intermating or further variety testing based on additive genetic and total genetic values. Selection of the top 5% progenies based on the multi‐trait index revealed positive genetic gains for fresh tuber yield (t ha^−1^), tuber yield (kg plant^−1^), and average tuber weight (kg). However, genetic gain was negative for tuber dry matter content and *Yam mosaic virus* resistance in comparison with standard varieties. Our results show the relevance of P‐BLUP for the selection of superior parental clones and progenies with higher breeding values for interbreeding and higher genotypic value for variety development in yam.

**Abbreviations**
LRT: log‐likelihood ratio test
P‐BLUP: pedigree estimated best linear unbiased prediction
REML: restricted maximum likelihood
YMV: *Yam mosaic virus*.

## 1 INTRODUCTION

Yam (*Dioscorea* spp.) is a clonally propagated monocotyledonous vine crop belonging to the genus *Dioscorea* in family Dioscoreaceae. This genus is composed of >600 species, of which 11 are economically important and widely cultivated for their starchy underground tubers or aerial bulbils (Coursey, 1967; Darkwa, Olasanmi, Asiedu, & Asfaw, 2020; Lebot, 2009; Siadjeu, Mayland‐Quellhorst, & Albach, 2018). Plants of *Dioscorea* species are mostly dioecious, with varying intraspecific and interspecific ploidy levels ranging from diploid to octoploid (2*n* = 40, 60, and 80) (Caddick, Wilkin, Rudall, Hedderson, & Chase, 2002; Darkwa, Olasanmi, Asiedu, & Asfaw, 2020; Mignouna, Abang, & Asiedu, 2008). They are highly heterozygous due to their obligate outcrossing (Tamiru et al., 2017).

Yam is produced on >8.5 million ha of land in about 61 countries distributed across tropical and subtropical Africa, Asia, Oceania, and Latin America (FAOSTAT, 2018). The world's annual yam production in 2017 was approximately 73 Tg, of which Africa accounted for 97% (FAOSTAT, 2018). White Guinea yam (*D. rotundata* Poir.) shows high importance for production, being the most planted and produced among cultivated species (Tamiru et al., 2017). It is indigenous to West Africa, a region that accounts for approximately 92% of global yam production (Asiedu & Sartie, 2010; FAOSTAT, 2018; Tamiru et al., 2017). In West Africa, yam is an integral part of the local food system, providing an estimated daily dietary calorie intake of >200 kcal for >300 million people (Alabi et al., 2019; Price, Bhattacharjee, Lopez‐Montes, & Fraser, 2018). Besides, yam in Africa has sociocultural relevance and is involved in many key life ceremonies (Lebot, 2009; Obidiegwu & Akpabio, 2017).

As for many other indigenous crops of Africa, the cultivation of white Guinea yam has not shown a yield increase in the last decades (Darkwa, Olasanmi, Asiedu, & Asfaw, 2020). Several biotic and abiotic stress factors, both on the field and in storage, significantly restrict its potential as a source of food and income. Some of these factors are increasing pressure of diseases and insect pests, the decline in soil fertility, and climate change‐related drought and heat stresses (Asiedu & Sartie, 2010; Obidiegwu & Akpabio, 2017). The International Institute of Tropical Agriculture (IITA) and its national partner institutions in West Africa have been working primarily on *D. rotundata* (2*n* = 40) since 1970 to improve crop quality and increase its value for the livelihoods of the present and future population. Their work comprises the development of resilient and productive yam varieties that respond well to the dynamics of current and future production challenges while meeting producer and consumer demand, which is a priority for ongoing research (Asiedu & Sartie, 2010; Darkwa, Olasanmi, Asiedu, & Asfaw, 2020).

Variety development is a cyclic and incremental process requiring traditional and new traits that respond well to the extremes of climate change and meet the rising demand for the crop as food, feed, and for industrial use. Choosing the right parents to create new genetic variants and selecting superior recombinants that have the desired features are among the critical action steps in the breeding process. Several economic traits are simultaneously considered when choosing parents for hybridization, as well as selecting the most favorable progenies that possess superior trait values for cultivation and consumption (Mondal, Hossain, Rasul, & Uddin, 2007; National Research Council, 1963). When selecting breeding plans, it is therefore essential to know the heritable variations and genetic correlations among traits of economic importance, and the expected occurrences of the desired progenies within the breeding population (Asfaw, Ambachew, Shah, & Blair, 2017). The assessment and prediction of such genetic parameters have extensively been used in crop breeding programs to optimize the breeding scheme and help to choose a breeding method to be used for genetic improvement (Falconer & Mackay, 1996). In yam, genetic parameter studies have primarily focused on dissecting the genetic variance within germplasm before the breeding for traits was undertaken (Agre et al., 2019; Alieu & Robert, 2014; Darkwa, Olasanmi, Asiedu, & Asfaw, 2020; Mignouna et al., 2008). However, this information has rarely been used for breeding purposes in yam (Darkwa, Olasanmi, Asiedu, & Asfaw, 2020).

This study aimed to assess genetic parameters accounting for additive and dominance effects in prediction for six essential traits in white Guinea yam breeding populations using pedigree information. The usefulness of integrating pedigree information in the assessment of plant performance in crop breeding trials has been demonstrated in many crops (Beeck, Cowling, Smith, & Cullis, 2010; Burgueño, de los Campos, Weigel, & Crossa, 2012; Cuevas et al., 2017, 2018; Pérez‐Rodríguez et al., 2015; Sukumaran, Crossa, Jarquin, Lopes, & Reynolds, 2017; Sukumaran, Crossa, Jarquín, & Reynolds, 2017). Incorporating genetic relationships based on pedigree information enhances the prediction accuracy in breeding trials, helping to identify the promising lines for commercial deployment, as well as select potential parents for future crosses (Oakey, Verbyla, Pitchford, Cullis, & Kuchel, 2006; Sukumaran, Crossa, Jarquin, Lopes, & Reynolds, 2017; Sukumaran, Crossa, Jarquín, & Reynolds, 2017). The information generated here can help to develop an effective strategy for white Guinea yam improvement.

**Core Ideas**

- Significant nonadditive genetic variances were found for yam traits.
- P‐BLUP is relevant for selection of parents and progenies in yam breeding.
- A multiple‐trait selection index has potential for identifying superior breeding lines.
- Model fit and prediction accuracy of breeding value vary with genetic effects and traits.

## 2 MATERIALS AND METHODS

### 2.1 Study materials and trial design

The study material comprised two sets of 83 full‐sib families represented by 1,420 progenies at the “second clonal generation” stage trial (see Darkwa, Olasanmi, Asiedu, & Asfaw, 2020 for details on the organization of yam breeding phases). The experiment was set in a row–column design with no repetitions, and pedigree information was used to estimate the variances and covariances among genotypes, allowing to predict breeding values and estimate genetic variances (Isik, Holland, & Maltecca, 2017). The first set was composed of 48 full‐sib families represented by 890 progenies obtained from crosses involving 17 female and 11 male parents. The second set was composed of 35 full‐sib families represented by 530 progenies obtained from crosses involving 14 female and 11 male parents. The two sets shared 11 female and seven male parental clones but differed in cross combinations. Families in the first set along with four standard varieties (local and improved) were grown at two sites—Ibadan (221 m altitude, 07°29.639′′ N, 003°54.092′′ E) and Abuja (431 m altitude, 09°09.842′′ N, 7°20.708′′ E), Nigeria—during the 2016 cropping season. The families in the second set, along with 11 standard varieties, were evaluated at Abuja during the 2017 cropping season. The trials were established using single‐row plots 3 m long and spaced 1 m apart. Three plants, spaced 1 m apart, were grown per plot. Planting was done in April at both sites. No fertilizer was applied. A mix of diuron 80 WG and glyphosate herbicide at 2.3 and 1.8 L ha^−1^, respectively, was applied after planting and before emergence to control the weeds. After plant emergence, the experimental field was kept free of weeds throughout the growth period of the plants by hand weeding. Harvesting was done 8 mo after planting. The details of the trial sites and design are presented in Supplemental Table S1.

### 2.2 Plant trait measurements

Eight plant traits (six main traits and two as a covariate) were assessed using the procedure described in yam trait ontology (http://www.cropontology.org/ontology/CO_343/Yam). The first trait was sett weight based on the weight of seed tubers or setts planted per unit area. The second trait was *Yam mosaic virus* (YMV) severity score based on a visual assessment on the relative area of plant tissue affected by yam mosaic virus disease. This trait was recorded as a proportion of plant surface affected using a five ordinal scale of 1–5 (1 = no visible virus symptom, 2 = mosaic on leaves, 3 = mild symptoms on few leaves but no leaf distortion, 4 = severe mosaic on most leaves and leaf distortion, and 5 = severe mosaic and bleaching with severe leaf distortion and stunting). The YMV scores were recorded on individual plants in a plot at 15‐d intervals in the trial at Ibadan and 30‐d intervals on trial at Abuja from 2 to 6 mo after planting. The timescale for YMV severity score data was used to calculate the progression curve for quantification of resistance. Other traits included the number of plants harvested per plot (count); the number of tubers per plant (count); tuber yield per plant (kg plant^−1^); average tuber weight (kg tuber^−1^); fresh tuber yield per hectare (t ha^−1^); and tuber dry matter content (%). Tuber yield per plant was determined as the weight of all fresh tubers divided by the number of plants harvested in a net plot. Average tuber weight was computed as the weight of all fresh tubers divided by the number of tubers harvested in a net plot. Fresh tuber yield per hectare was determined as the weight of all the fresh tubers harvested per plot multiplied by 10, and the result divided by the area of effective plot in square meters. The weight of all fresh tubers harvest from a net plot of size 3 m^2^ was measured in kilograms. To estimate dry matter content, 200 g of fresh tuber was chopped into small pieces and oven dried at 70 °C for 24–48 h until a constant weight was achieved. Dry matter content was determined as

$$\mathrm{DM}\;\left(\%\right)=\left(\frac{\mathrm{dry}\;\mathrm{weight}}{\mathrm{fresh}\;\mathrm{weight}}\right)\times \;100$$

### 2.3 Data analyses

Data were subjected to different analyses to assess population structure, to estimate the relative genetic variability, as well as to predict the genetic values of parents and their progenies in the breeding population. The discriminant analysis of principal components (DAPC) was used on the pedigree relationship matrix to investigate the genetic structure using adegenet package (Jombart, Devillard, & Balloux, 2010) in the R environment (R Core Team, 2019). The find.clusters and optim.a.score functions in adegenet package were used to infer genetic clusters and determine the optimum number of principal factors to be retained for analysis. The eigenvalues associated with the first five principal components cumulatively accounted for >77% of the fraction of total genetic variation and were added in the statistical model as fixed covariates to correct the population structure effect (Azevedo et al., 2017; Enciso‐Rodriguez, Douches, Lopez‐Cruz, Coombs, & de los Campos, 2018). Three alternative one‐step linear mixed models that used either identity matrix, additive matrix, or additive plus dominance matrices were investigated and compared. The models were fit independently for each trait using the average information (AI) restricted maximum likelihood (REML) algorithm (Gilmour, Thompson, & Cullis, 1995) in the ASReml‐R version 4 package (Butler, Cullis, Gilmour, Gogel, & Thompson, 2018). The following baseline model (B) that assumes no genetic relationship among clones (i.e., identity matrix) was used for initial estimates of the genetic effects for the trait (response variable):

$$Y_{ij}=\;\mu +\sum_{i\;=\;1}^{n}PC_{hi\gamma h}+N_{j}+b_{j}+g_{i\left(j\right)}+\varepsilon_{ij}$$

where $Y_{ij}$ is the vector of the phenotypic variable across *j* trials. The trials at Ibadan 2016, Abuja 2016, and Abuja 2017 constituted three trials making *j* = 3, μ is overall mean, $\sum_{i\;=\;1}^{n}PC_{hi\gamma h}$ is a regression on eigenvalues associated with the optimum number of pedigree‐derived principal components (*n* = 5) cumulatively accounted for a substantial fraction of the total genetic variation. $N_{j}$ is a fixed trial effect. $b_{j}$ is a trial‐specific design‐based field blocking structure effect, $g_{i}$ is the main effect of clone (genotype) within trial, and $\varepsilon_{ij\;}$is a random error within each trial. The direct sum structure for the residual error term was fitted. Sett weight (weight of seed tubers used for planting) and the number of plants at harvest were added to the model as covariates for tuber yield characteristics whenever significant using a Wald test. The plot $b_{j}∼N\left(0,\;I\sigma_{b}^{2}\right)$, clone main $g_{i}∼N\left(0,\;I\sigma_{g}^{2}\right)$, and random error $\varepsilon_{ij}∼N\left(0,\;I\sigma_{\varepsilon }^{2}\right)$ effects where **I** is an identity matrix. $\sigma_{b}^{2}$, $\sigma_{g}^{2}$, and $\sigma_{\varepsilon }^{2}$ are between‐plot, clone main, and within‐plot error variances, respectively.

Aside from the baseline model (B) that assumes no genetic relatedness among clones ($Vg_{i}=I\sigma_{g}^{2}$), two genetic models with additive (A) and additive plus dominance (A+D) covariance structures from the pedigree matrix for the clone main effects within trial $\left(g_{i}\right)$ were tested. The total genetic variance was then partitioned to the additive, dominance, and residual genetic effects following the approach implemented in various studies (Beeck et al., 2010; Endelman et al., 2018; Oakey et al., 2006; Ovenden, Milgate, Wade, Rebetzke, & Holland, 2018). For the additive model, the clone main effect within trial $\left(g_{i}\right)$ was decomposed into additive genetic $a∼N(0,\;A{\varphi \sigma }_{a}^{2})$ and residual genetic $\hat{g}∼N\left(0,\;I\sigma_{\hat{g}}^{2}\right)$ within‐trial effects, such that $g_{i}=\;a+d+\hat{g}$. For the A+D model, the clone main effect within trial $\left(g_{i}\right)$ was partitioned into additive, dominance $d∼N(0,\;D{\varphi \sigma }_{d}^{2})$, and residual genetic effects, such that $g_{i}=\;a+d+\hat{g}$. $\sigma_{a}^{2}$, $\sigma_{d}^{2}$, and $\sigma_{\hat{g}}^{2}$ are additive, dominance, and residual genetic variances, respectively. The differences among the additive, dominance, and residual genetic effects are that the additive and dominance effects have a covariance structure proportional to additive and dominance genetic relationships derived from the pedigree, respectively, whereas the residual genetic effect corresponds to the independent clone effect (Oakey et al., 2006; Beeck, Cowling, Smith, & Cullis, 2010). *A*φ and *D*φ are the pedigree co‐ancestry coefficients for additive and dominance matrices, respectively, computed with package AGHmatrix using a diploid option (Amadeu et al., 2016) in R version 3.6 (R Core Team, 2019). The goodness of model fit was assessed by REML log‐likelihood ratio test (LRT) (Lewis, Butler, & Gilbert, 2011). The LRT compared the log‐likelihoods of models which differ in their random effects using a χ^2^ test.

The variance components accounted for each of the terms included in the model were estimated for the measured traits (see Supplemental Table S2) using packages asremlPlus and ASReml‐R 4 (Brien, 2020; Butler et al., 2018). The total phenotypic variance was partitioned into between‐plot ($\sigma_{b}^{2}$) effect, clone main effect ($\sigma_{g}^{2}$) that associated with additive effect ($\sigma_{a}^{2}$), dominance effect ($\sigma_{d}^{2}$), and residual genetic effect ($\sigma_{\hat{g}}^{2}$) and within‐plot error ($\sigma_{e}^{2}$) variances that varied according to the model. Other parameters such as coefficient of determination of plot effect ($C_{b}^{2}$), coefficient of genotypic (total genetic) variation (CV_g_), coefficient of phenotypic variation (CV_p_), residual coefficient of variation (CV_e_), and coefficient of relative variation (CV_g_/CV_e_) were calculated for the best fit model. Correlations among the genetic effect of the traits were assessed using GGally package in R (Schloerke et al., 2020).

The broad‐sense (*H* ^2^) and narrow‐sense (*h* ^2^) heritability estimates for each trait were calculated as $h^{2}=\sigma_{a}^{2}/\left(\sigma_{g}^{2}+\sigma_{b}^{2}+\sigma_{e}^{2}\right)$ and $H^{2}=\sigma_{g}^{2}/\left(\sigma_{g}^{2}+\sigma_{b}^{2}+\sigma_{e}^{2}\right)$, respectively. Heritability was categorized as low (0–30%), moderate (31–60%), and high (>60%) according to (Robinson, Comstock, & Harvey, 1949). The coefficients of genetic variation, phenotypic variation, and the residual error coefficients were determined following the formula described in Burton and DeVane (1953) as

$$CV_{g}\;=\frac{\sqrt{\sigma_{g}^{2}}}{\bar{x}}\;\times \;100$$

$$CV_{p}\;=\frac{\sqrt{\sigma_{p}^{2}}}{\bar{x}}\;\times \;100$$

$$CV_{e}\;=\frac{\sqrt{\sigma_{e}^{2}}}{\bar{x}}\;\times \;100$$

where $\sigma_{g}^{2}$, $\sigma_{p}^{2}$, and $\sigma_{e}^{2}$ are variances for total genetic, phenotypic, and residual error components, respectively, for the trait from the best fit model in REML analysis, and $\bar{x}$ is the trait mean value.

Genotypic (total genetic) values (GV*_i_*) were computed as the sum of main effects for **a**, **d**, and $\hat{g}$, whereas breeding values (BV*_i_*) were computed as the main effect for **a** (additive genetic) for an individual *i* from the best fit model, following the approach implemented in Ovenden et al. (2018). The theoretical accuracy of breeding values estimation was assessed using the formula (Isik et al., 2017; Suontama et al., 2019):

$$r\;=\sqrt{1-\frac{\mathrm{PE}V_{i}}{A_{ii}\sigma_{a}^{2}}}\;$$

where PEV*_i_* is prediction error variance (Mrode, 2014) of individual *i* and $A_{ii}$ is the diagonal element of the pedigree‐based relationship matrix for the *i*th individual correcting additive genetic variance $\left(\sigma_{a}^{2}\right)$ for inbreeding.

In ranking and selection of superior individuals for genetic improvement and gain for multiple traits simultaneously, the following additive selection index for an estimated breeding or genotypic values was used (de Figueiredo, Airton, Nunes, & Borges, 2012):

$$\;I_{i}=\sum_{k=1}^{n}{\hat{\ddot{g}}}_{it}\times \mathrm{wt}\times \frac{1}{{\hat{\sigma }}_{gt}}\;$$

where *I_i_* is the index value associated with estimated breeding value or genotypic value of an individual *i*; ${\hat{\ddot{g}}}_{it}$ is the P‐BLUP‐estimated breeding or genotypic value of an individual *i* for trait *t*; wt is the presumed economic weight or proportional importance associated with trait *t*; and ${\hat{\sigma }}_{gt}$ is additive genetic or genotypic standard deviation for trait *t*. The economic weights assigned were 0.35 for total fresh tuber yield (t ha^−1^), tuber yield per plant (kg plant^−1^), average tuber weight (kg), and tuber dry matter (%), 0.2 for tuber number per plant, and −0.14 for YMV (area under the disease progress curve [AUDPC] value). Aiming to generate future populations combining higher trait values, a subset of clones was selected with a 5% selection intensity based on the ranking obtained with the model described. Likewise, the additive selection index of the breeding values of the parental clones based on progeny performance was calculated and used as a backward selection to identify superior progenitor for further use in the breeding program.

The genetic gain per cycle (ΔG) or time ($\Delta G_{t}$) and a percentage of the population mean ($\Delta G_{m}$) were estimated to assess the expected genetic gain for traits considered for selection of superior clones using the procedure elaborated by Cobb et al. (2019) as

$$\Delta G=K\times \sqrt{h^{2}}\times \sigma_{a}$$

$$\Delta G_{t}=\frac{K\times \sqrt{h^{2}}\times \sigma_{a}}{\mathrm{time}}$$

where *K* is the standardized selection differential, which is 2.06 at 5% intensity, $\sqrt{h^{2}}$ is the repeatability or precision of additive breeding value estimation (also called selective accuracy), $\sigma_{a}\;$ is the additive genetic standard deviation for the trait, and time is cycle length, which is 4 yr.

$$\Delta G_{m}=\frac{K\times \sqrt{h^{2}}\times \sigma_{a}}{\bar{x}}\;\times \;100$$

The $\Delta G_{m}$ values were classified as low (<10%), moderate (10–20%) and high (>20%), as described by Johnson, Robinson, and Comstock (1955). The genetic gain expected on measured traits was assessed taking standard cultivars as a benchmark using the formula

$$\Delta G_{c}=\frac{{\bar{X}}_{it}\;-\;C_{it}}{C_{it}}\;\times \;100$$

where $\Delta G_{c}$ is observed genetic gain as compared with the standard cultivars, ${\bar{X}}_{it}\;$is the average of the BLUP values of the selected individuals for trait *t*, and $C_{it}$ is best linear unbiased prediction values of the respective standard (improved or local) cultivars. $\Delta G_{c}$ was used to quantify the genetic gain for the selection performed using the breeding values of the best 5% clones with a multiple‐trait index.

## 3 RESULTS

**FIGURE 1:**
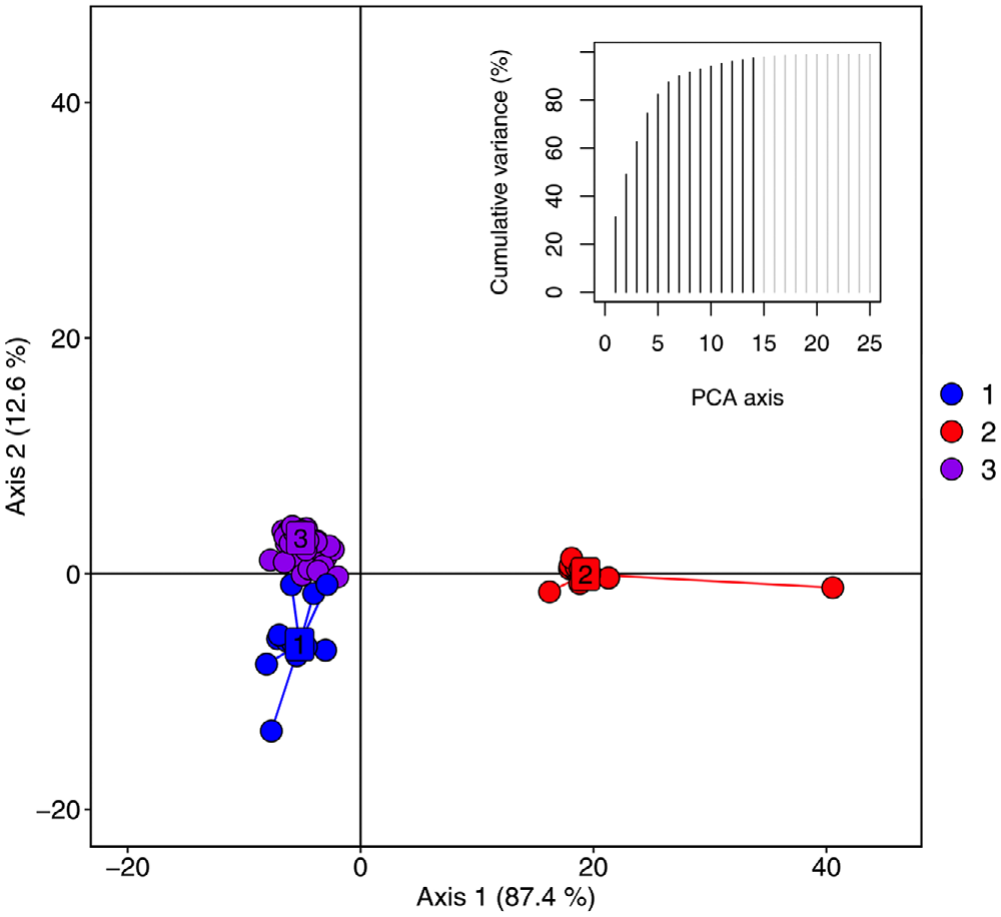
Clustering of the 1,420 yam breeding lines with discriminant analysis (DA) of principal components on pedigree‐based relationship matrix. PCA, principal component analysis

Discriminant analysis of principal component (DAPC) on the pedigree‐based relationship matrix revealed population structure among the yam clones (Figure 1). The first 14 principal components were found optimum to capture the population structure arising from genetic relatedness among the yam genotypes. Among these, the eigenvalues associated with the first five principal components accounted for >77% of the genetic variation. The genotype grouping has justified pre‐correcting the phenotypic records of traits for the effects of genetic structure in the prediction models.

### 3.1 Variance components

Variance component estimates for the six yam traits from three alternative models are presented in Supplemental Table S2. Adding either the additive or dominance variance–covariance structures into the baseline model was significant to describe the variances for all yam traits (Table 1). However, the optimal fit was obtained by including additive effect alone in the model for average tuber weight, tuber number per plant, and tuber dry matter. Including dominance in the model had a significant effect for tuber yield (per unit area or per plant) and YMV severity.

**TABLE 1:** Log‐likelihood ratio test (LRT) comparing the goodness of fit for genetic models (A and A+D^a^) using variance–covariance structure from a pedigree relationship on six yam traits relative to a baseline model (B) with an independent clone effect

	LRT value^b^					
Model	TTY	TTWPL	ATW	TTNPL	DM	YMV
B vs. A	7.02**	5.63	28.51***	34.317***	24.02***	15.82***
B vs. A+D	19.84***	13.95***	31.496***	39.47***	25.54***	25.55***
A vs. A+D	12.82***	8.316***	2.98^†^	5.15	1.52	9.72***

^a^ A, model fitted with additive variance–covariance structure; A+D, model fitted with additive plus dominance variance–covariance structure.

^b^ TTY, fresh tuber yield; TTWPL, fresh tuber yield per plant; ATW, average tuber weight; TTNL, tubers per plant; DM, tuber dry matter content; YMV, *Yam mosaic virus* severity.

^†^Significant at the .10 probability level. ^**^Significant at the .01 probability level. ^***^Significant at the .001 probability level.

**FIGURE 2:**
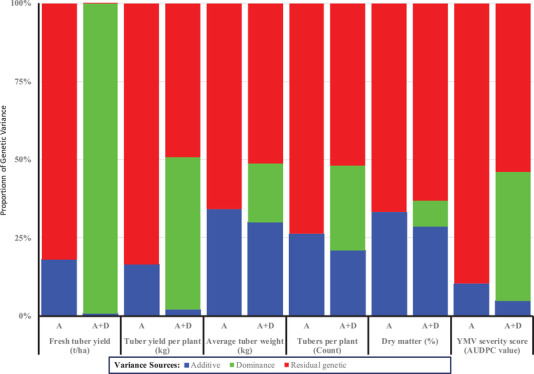
Genetic variance partitioning of six white Guinea yam traits in 1,420 early‐generation breeding lines for the genetic models accounting for different covariance structures from a pedigree relationship. A, additive model. A+D, additive plus dominance model; YMV, *Yam mosaic virus*; AUDPC, area under the disease progress curve

The proportion of total genetic variance due to additive genetic component ranged from 10 (YMV severity score) to 34% (average tuber weight) with the A model (Figure 2). Inclusion of the dominance effect caused a reshuffling of the genetic variance components with a drop in additive genetic variance for all measured traits. The additive variance was reduced by 12–95% of total genetic variance, depending on the trait with the inclusion of the additive and dominance (A+D) covariance structure to the baseline model. In contrast, the residual genetic variance represented the highest proportion (∼60%) of the total genetic variance for tuber dry matter with the A+D model. The variances for the genotypic effect were higher than the random error effect for all measured traits, with the coefficient of relative variation ranging from 1.1 for fresh tuber yield (t ha^−1^) to 4.45 for YMV severity (Table 2). The coefficient of determination of the plot effect ($C_{b}^{2}$) was low (<0.11) for all the traits. The error coefficient variation (CV_e_) was relatively low, ranging from ∼8% for YMV severity to 29% for tuber yield per plant. The coefficients of genetic and phenotypic variations (CV_g_ and CV_p_) were moderate to high for all measured traits. Yam mosaic virus severity score and tuber dry matter content had medium (between 10 and 20%) values of the genotypic and phenotypic coefficient of the estimated variances. In comparison, these values were higher (between 28 and 54%) for other traits.

**TABLE 2:** Estimates of genetic parameters for fresh tuber yield, tuber yield per plant, average tuber weight, tubers per plant, tuber dry matter content, and *Yam mosaic virus* (YMV) severity (area under the disease progress curve [AUDPC] value) in 1,420 white Guinea yam early‐generation clones evaluated at three sites

	Trait					
Estimates of parameters^a^	Fresh tuber yield	Tuber yield per plant	Avg. tuber weight	Tubers per plant	Tuber dry matter	YMV severity score
	t ha^−1^	kg	kg tuber^−1^	no.	%	AUDPC value
Mean	16.25 ± 4.01	1.78 ± 0.46	1.28 ± 0.45	1.51 ± 0.21	32.88 ± 3.67	228.89 ± 29.14
$\sigma_{g}^{2}$	21.13 ± 5.03	0.33 ± 0.06	0.30 ± 0.06	0.21 ± 0.03	15.59 ± 2.04	1,389.46 ± 111.05
$\sigma_{b}^{2}$	3.31 ± 1.45	0.03 ± 0.02	0.03 ± 0.02	0.01 ± 0.03	3.11 ± 0.75	47.93 ± 21.05
$\sigma_{e}^{2}$	19.19 ± 3.94	0.19 ± 0.05	0.14 ± 0.05	0.16 ± 0.03	8.78 ± 1.45	312.18 ± 76.77
$\sigma_{p}^{2}$	43.63 ± 2.56	0.56 ± 0.03	0.47 ± 0.03	0.37 ± 0.02	27.48 ± 1.58	1,749.58 ± 80.92
$C_{\mathrm{plot}}^{2}$	0.08	0.05	0.06	0.03	0.11	0.03
CV_g_, %	28.29	32.32	43.20	30.35	12.01	16.29
CV_p_, %	40.65	42.04	53.56	40.28	15.94	18.27
CV_e_, %	26.96	24.81	28.14	26.49	9.01	7.72
CV_r_	1.10	1.74	2.14	1.31	1.78	4.45

^a^ $\sigma_{g}^{2}$, genetic variance; $\sigma_{b}^{2}$, environmental variance between plots within experiments; $\sigma_{e}^{2}$, within‐plot error variance; $\sigma_{p}^{2}$, individual phenotypic variance, which is sum the variance components for the trait; $C_{\mathrm{plot}}^{2}$, coefficient of determination of plot effect; CV_g_, coefficient of genotypic variation; CV_e_, residual coefficient of variation; CV_r_, coefficient of relative variation $\left(\sigma_{g}^{2}/\sigma_{e}^{2}\right)$.

**FIGURE 3:**
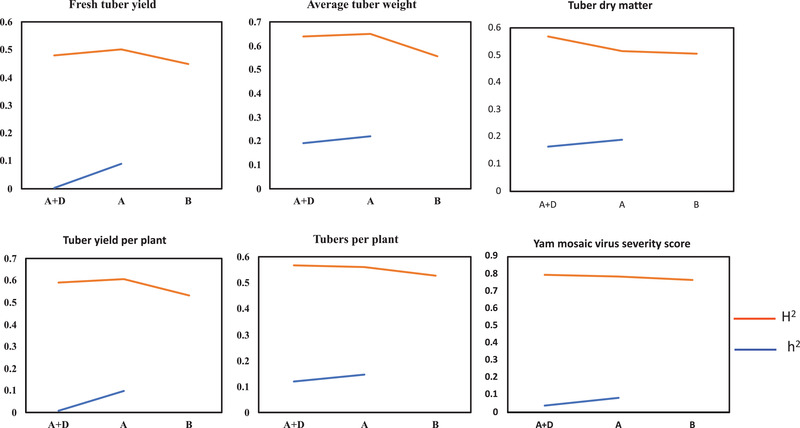
Variation in heritability estimates (broad sense and narrow sense) with three different models for the six traits in white Guinea yam. A, genetic model with additive covariance structure from the pedigree matrix; A+D, genetic model with additive plus dominance covariance structure from the pedigree matrix; B, baseline model with independent clone effect. *H* ^2^ is broad‐sense heritability. *h* ^2^ is narrow‐sense heritability

Both narrow‐ and broad‐sense heritability estimates varied among the traits and for the different models (Figure 3, Supplemental Table S2). The narrow‐sense heritability estimates gradually decreased with the addition of the dominance effect in the model. The broad‐sense heritability estimates, however, generally improved with fitting the genetic variance–covariance structure into the baseline model for all traits except the YMV severity score (Figure 3). For YMV, broad‐sense heritability remained unchanged across the different models. Accuracy of estimated breeding values with the additive and dominance effects varied in yam traits. The additive effects model has resulted in gain in breeding value accuracy for tuber number per plant, tuber weight per plant, average tuber weight, and tuber dry matter. A marginal gain in breeding value accuracy was achieved with the dominance effects for fresh tuber yield (t ha^−1^) and YMV (Supplemental Figure S3).

**FIGURE 4:**
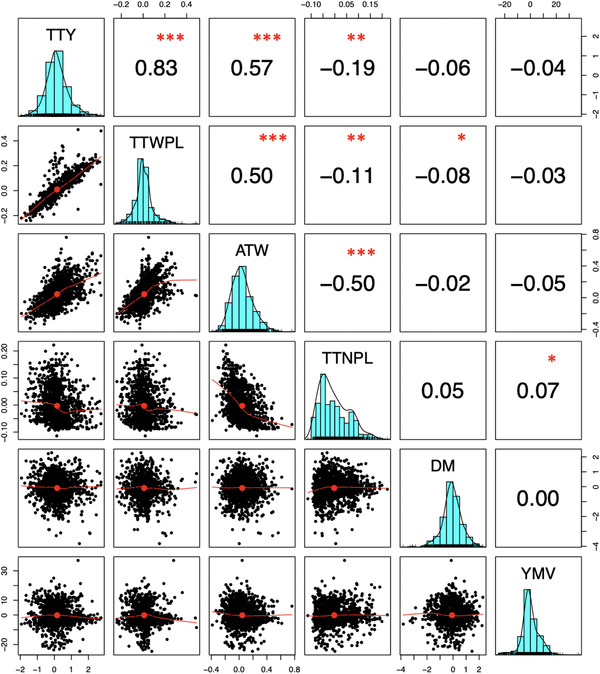
The pattern of genetic correlations among the six white Guinea yam traits. Statistical significance is labeled ^*^ *p* < .05, ^**^ *p* < .01, ^***^ *p* < .001. TTY, fresh tuber yield (t ha^−1^); TTWPL, fresh tuber yield per plant (kg); ATW, average tuber weight (kg tuber^−1^); TTNPL, tuber number per plant (count); DM, tuber dry matter content (%); YMV, *Yam mosaic virus* severity score (area under the disease progress curve [AUDPC] value)

The genetic correlations among the measured traits varied in degree and direction (Figure 4). The estimated genetic relationships among tuber yield traits (fresh tuber yield [t ha^−1^], tuber yield kg per plant, and average tuber weight) were strong and positive (*P* < .001). On the other hand, the tuber number had strong and negative estimated genetic correlations with average tuber weight, fresh tuber yield per plant, and unit area. The correlations of genetic values of tuber dry matter were negative with all measured traits except positive but weak with tuber number per plant. Yam mosaic virus severity score had weak associations with all measured traits for the genetic effects.

### 3.2 Selection options for establishing base, breeding, and deployment populations

**FIGURE 5:**
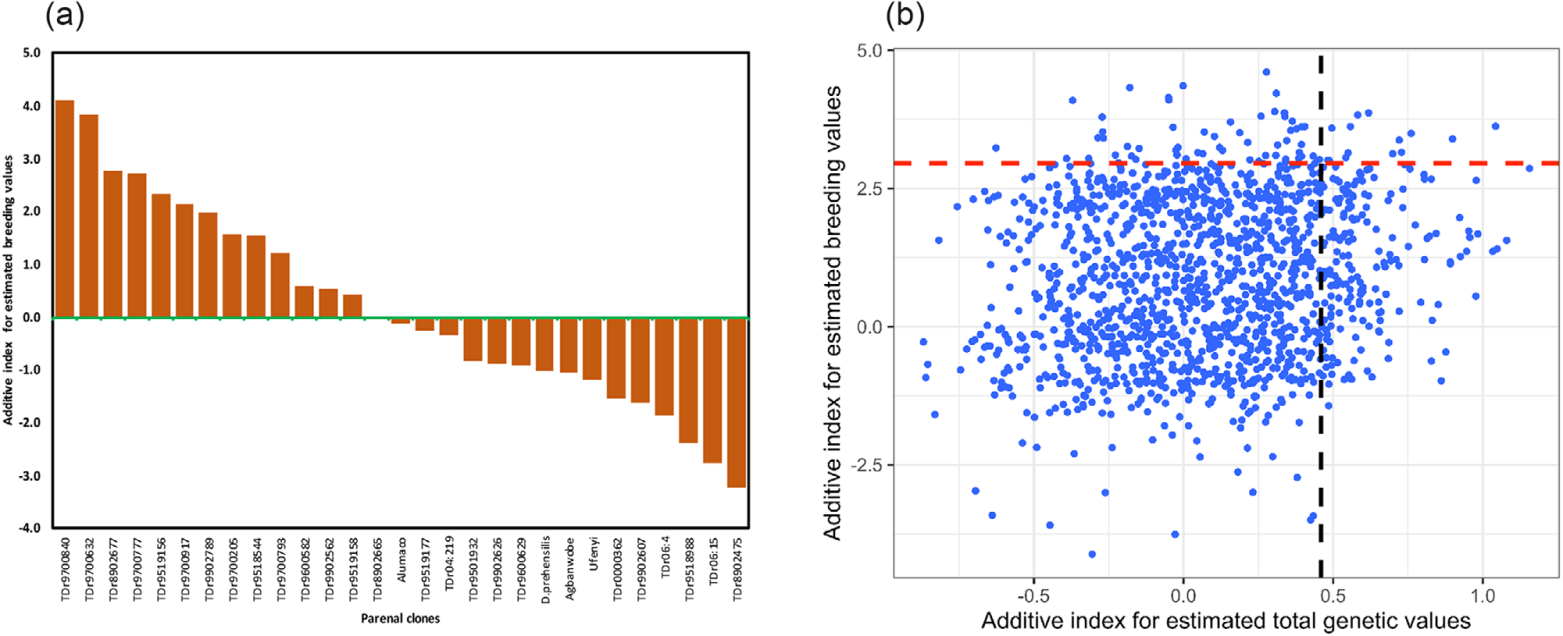
Distribution of the parental clones and their progenies based on the breeding and genotypic values estimated through the restricted maximum likelihood (REML) procedure involving pedigree information. (a) Parental clone ranking on net genetic worth for the multiple trait selection indices for breeding value estimates based on progeny performance, (b) distributions of the clonal progenies for selection index on net breeding and genotypic values for multiple traits. The graph in Panel b presents individual progeny genotypes as blue dots. The red and black dotted lines represent the cut‐point for selection index at 5% intensity

The ranking of parental clones and their clonal progenies for their net genetic merits on a multi‐trait selection index was obtained (Figure 5). The multiple‐trait index for the breeding values identified 13 clones having a higher genetic capacity for simultaneous improvement of the studied traits in yam breeding (Figure 5a). However, the parental clones with superior genetic merits for multiple selection index expressed both positive and negative breeding values for the individual traits (data not shown). Out of the 13 parental clones with additive genetic value net worth for the traits considered in selection index, 12 had positive breeding values for fresh tuber yield, 13 for tuber yield per plant, 9 for average tuber weight and tuber number per plant, 4 for tuber dry matter, and 10 for YMV tolerance. The 13 clones are potential parents for incorporation into the selection list for establishing a new base population in the breeding program.

**FIGURE 6:**
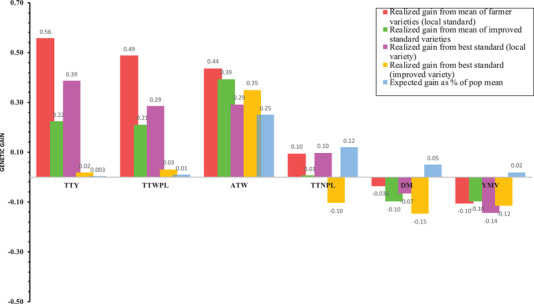
Comparison of realized and expected genetic gains for measured traits with multi‐trait index and single‐trait selection strategies. YMV, *Yam mosaic virus* severity (area under the disease progress curve [AUDPC] value); TTNL, tubers per plant (count); TTWPL, fresh tuber yield per plant (kg); TTY, fresh tuber yield (t ha^−1^); ATW, average tuber weight (kg); DM, tuber dry matter content (%)

We also assessed the genetic merits of the clonal progenies based on an index that simultaneously considered the respective breeding and genotypic values of the six measured traits (Figure 5b). The progenies that fell above the horizontal red dotted lines (cut‐points for selection at 5% intensity) had the best index for the breeding values. In contrast, those at the left of the vertical black dotted line showed useful indices of genotypic values for the measured traits. Few progenies at the right corner (above the red dotted and at the left of black dotted lines) of the graph (Figure 6b) combined the potential as an excellent parent in crosses and candidates for future release as a new variety in the breeding program. The top 5% clonal progenies for breeding value estimate (above red dotted lines) are potential parents for recycling in the breeding program targeting simultaneous improvement for the traits.

Table 3 and Figure 6 compare the relative selection gain by clonal progeny selection for the measured traits with different methods. The estimates of expected genetic gain were generally low (<10% of population mean) for tuber yield per unit area, tuber yield per plant, tuber dry matter, and YMV severity score. At the same time, it was moderate for tubers per plant and high for average tuber weight. The values in Figure 6 compared the realized and expected genetic gain of measured traits with single trait and multi‐trait selection objectives. Response to multi‐trait index selection presented gain in percentage relative to the local and improved standard varieties. Conversely, expected genetic gain with single trait selection assessed the improvement for the trait in the mean value of selected individuals over the population mean.

**TABLE 3:** Expected genetic gain based on 5% selection differential for the six white Guinea yam traits assessed in early‐generation evaluation trials

Character	ΔG	$\Delta G_{t}$
Fresh tuber yield (t ha^−1^)	0.05	0.01
Fresh tuber yield per plant, kg	0.02	0.01
Avg. tuber weight. kg tuber^−1^	0.31	0.08
Tuber no. per plant	0.19	0.05
Tuber dry matter, %	1.77	0.44
YMV severity score, AUDPC value	3.32	0.83

*Note*. ΔG, genetic gain per cycle; $\Delta G_{t}$, genetic gain per year; YMV, *Yam mosaic virus*; AUDPC, area under the disease progress curve.

The top 5% progenies based on multiple selection index showed a positive genetic gain over both local and improved standard varieties for fresh tuber yield (t ha^−1^), tuber yield per plant (kg), and average tuber weight (kg tuber^−1^). However, such gain was negative over the outstanding standard variety (improved) for tubers per plant and all types of benchmark varieties for tuber dry matter and YMV severity score. The response to multi‐trait index selection for tuber dry matter and YMV severity score was not in the desired direction. The mean tuber dry matter content of the selected individual in comparison with the premier standard variety was lowest. The mean YMV severity score for the top 5% selected individuals was higher than that of the outstanding standard cultivar in the trial. However, three clonal progenies in the selection list outperformed the standard varieties in response to YMV infestation (data not shown). For tuber dry matter, none of the individuals in the selection list exceeded the leading standard cultivar.

## 4 DISCUSSION

Better understanding of the genetic control of economic traits in breeding populations is vital for a breeding program to develop productive and resilient varieties with acceptable end‐user qualities. In this study, pedigree information was used to estimate the variance and covariance among the genotypes and predict genetic merits for tuber yield, quality, and disease traits in white Guinea yam breeding populations. The variance estimation and prediction of genetic values in the current set materials provided useful insights to initiate a new breeding population while attaining genetic gain by selecting superior clonal progenies. Our results showed that the six yam traits studied are all responsive to selection based on breeding value, but to varying degrees.

Breeding value alludes to the property of an individual in a population that is attributable mainly to the additive genetic variance of a trait (Falconer & Mackay, 1996). The variance in additive genetic value is the fraction of total genetic variance of a quantitative trait that is explained by the sum of effects of different alleles; therefore is transmittable from parents to offspring and relevant to the selection response (Enciso‐Rodriguez et al., 2018). The variance component values obtained in our study indicated that the additive effects with the A model were higher than those obtained with the A+D model for all the six yam traits (Figure 2). However, inclusion of the dominance effect explained part of the estimated genetic variance in the model (A+D) significantly decreased the fraction of additive variances and, consequently, the narrow sense heritabilities (Figure 3). Goodness‐of‐fit test with LRT showed that inclusion of the dominance effect improved the model fit slightly for three traits out of the six considered in our analysis. The improvement in the model fit when accounting for dominance, however, did not translate into significant gain in prediction accuracy of the estimated breeding values except for the YMV severity. For YMV, though the size of the additive effect was small in the A model compared with the A+D model, the prediction accuracy improved with the latter. Likewise, variation among the plant traits in model fit and prediction accuracy was reported in potato (*Solanum tuberosum* L.) with the inclusion of additional genetic covariance structure into the additive model (Amadeu et al., 2020; Enciso‐Rodriguez et al., 2018; Endelman et al., 2018). According to these authors, prediction ability with nonadditive effects was not superior to that obtained with the additive model. The estimates of prediction accuracies for the yam traits in our study were generally high, which could be attributable to the lack of pedigree depth information in yam and overall bias with the pedigree‐based methods (de Bem Oliveira et al., 2019). Our results on yam generated new insights to supplement the previous findings in other crops like potato that adding dominance effects probably does not improve the prediction accuracy of all plant traits. However, gain in prediction accuracy with the dominance model is achieved on traits like disease resistance that are normally controlled by fewer genes (Amadeu et al., 2020). Some of the dominance effects were absorbed in the additive effect in a model that considered additive covariance structure alone from the pedigree relationship in yam. This lack of orthogonality in variance decomposition could be attributable to the pedigree‐based method limitations to capture the Mendelian segregation in a breeding population, which often deviates from the Hardy–Weinberg equilibrium (HWE) and linkage equilibrium (LE). Our breeding population composed of full and half‐sib families developed from selective mating and subjected to directional selection. The numbers of female and male parents are not equal, and not all parents are crossed with each other in the population used by our study. Moreover, our breeding population in this study was subjected to continuous improvement through generations of directional selection that make them deviate of HWE. In this context, the pedigree‐based method lacks the power to dissect genetic relationships effectively among individuals in the study population and precisely partition the genetic variances (Muñoz et al., 2014; de Bem Oliveira et al., 2019). It also presents low efficiency in capturing the genetic similarity of founders in the pedigree and in distinguishing variances within families (de Bem Oliveira et al., 2019). The interdependence between the additive and nonadditive genetic variances has also been reported in other studies (Bouvet, Makouanzi, Cros, & Vigneron, 2015; Muñoz et al., 2014; Xiang, Christensen, Vitezica, & Legarra, 2018).

Any combination of the genetic factors that results in superior genotype is helpful for improvement through direct selection in clonally propagated crops like yam. The moderate to high (0.45–0.79) broad‐sense heritabilities of the focus traits in this study suggested the expectation for genetic improvement with clonal selection as highly positive. However, the estimates of expected genetic gain as a percentage of population means are low for all the measured traits except average tuber weight and tuber number per plant (Figure 6). The expected genetic gain estimates suggest improving the source population through the crossing of individuals with high additive genetic values to drive genetic gain in the current set of materials. It is only the additive genetic effects that are transmitted to offspring via sexual reproduction.

The pattern of interrelation among the genetic effect for the traits justifies considering the selection index to improve the source population for multiple characteristics simultaneously (Figure 4). Breeding programs often select for several characters and use different selection options: tandem selection, independent culling, and index selection (Acquaah, 2007; Bos & Caligari, 2008; Falconer & Mackay, 1996). The P‐BLUP/REML analysis of progeny data implemented in this study enabled the identification of superior parental clones based on their progeny performance (backward selection), as well as the choice of best‐performing clonal progenies with outstanding potential for use as parents (forward selection) via a multi‐trait selection index. Considering that only the additive effects are transmitted to offspring via sexual reproduction, the ranking of progenitors according to their progeny performance is highly essential. For many of the studied traits, the narrow‐sense heritabilities are low. Hence, the progeny test allows the selection of superior progenitors to generate a new base breeding population and drive genetic gain in characters with low heritability values (Falconer & Mackay, 1996; Hill & Mackay, 2004).

Applying a multi‐trait selection index on the clonal progenies led to the identification of a subset of superior clones with high breeding values. A higher gain in selection for multiple‐trait breeding values compared with the single‐trait, especially when traits are negatively correlated (Bauer & Léon, 2008). Also, Ojulong, Labuschagne, Herselman, and Fregene (2010) and Missanjo and Matsumura (2017), in their respective studies, identified superior progenies for economically important traits using an index based on multiple traits.

The selection for multi‐trait breeding values identified 71 clonal progenies with great potential for the simultaneous improvement for the measured traits. Crosses involving these selected clones from the genetically different groups (Figure 1) are expected to increase the frequency of favorable alleles in the resulting progenies while maximizing genetic variability and heterosis. The genetic gain observed from selecting the best 5% of the progenies based on the multi‐trait index for breeding values could provide a small glimpse or general overview of the progress achieved in yam breeding (Figure 6). Based on this premise, the positive genetic gain over standard varieties for fresh tuber yield (t ha^−1^), tuber yield (kg plant^−1^), and average tuber weight (kg tuber^−1^) could indicate progress achieved for these traits in the breeding program. For tuber dry matter content and YMV resistance, not much progress has been attained in the white yam breeding program. Hence, the conventional selection method will be inadequate to enhance genetic gain for these two traits. Indeed, new trials or studies are required to accurately estimate the realized genetic gain for the various traits in the yam breeding program.

Other suggestions to enhance genetic gain for such traits in clonally propagated crops include heterosis exploiting breeding schemes, or population hybrid breeding (Grüneberg et al., 2009, 2015). Significant genetic gain reported using such breeding schemes in sweetpotato [*Ipomoea batatas* (L.) Lam.] for dry root yield, as well as recessively inherited traits such as resistance to sweet potato virus disease and sugar content (Grüneberg et al., 2015). Their results suggest that the simultaneous use of heterosis exploiting breeding schemes and the reciprocal recurrent selection was expected to result in a steep increase in genetic gain.

Overall, this study has provided valuable insights into the genetic control and genetic gain, as well as a glimpse of the status of the six essential traits in the white Guinea yam breeding program. Parental and progeny clones identified through backward and forward selection, respectively, with a high and positive index could be used as potential trait progenitors to generate progenies with good agronomic and end‐product quality in white Guinea yam. Hybridization of genetically different clones identified from the top 5% of superior progenies is expected to increase the frequency of favorable alleles while maximizing genetic variability. The superior clones having higher genotypic values identified through the multi‐trait selection index should be tested further for possible commercial deployment.

## AUTHOR CONTRIBUTIONS

Asrat Asfaw: Conceptualization; Formal analysis; Supervision; Writing‐original draft; Writing‐review & editing. Bunmi Olasanmi, and Ayodeji Abe: Supervision; Writing‐review & editing. Patrick Adebola, and David De Koeyer: Project administration; Writing‐review & editing. Kwabena Darkwa, Dotun Samuel Aderonmu & Paterne Agre: data collection; trial management; Writing‐review & editing. Robert Asiedu: Funding acquisition; Writing‐review & editing

## CONFLICT OF INTEREST

The authors declare that there is no conflict of interest.

## Supporting information

Supplemental Table S1. Details of the field experiments.

## Data Availability

Data used for this article is available at www.yambase.org and will also be supplied upon request to the corresponding author.
