# Supplementary material for: Genetic parameters, prediction, and selection in a white Guinea yam early‐generation breeding population using pedigree information
Source: Crop Sci. 2020 Dec 22;61(2):1038–51. doi: 10.1002/csc2.20382 (PMC8048640; doi:10.1002/csc2.20382)
Supplement: Supplementary file 1 — Supplemental Table S1. Details of the field experiments. [file CSC2-61-1038-s001.docx]

**SUPPLEMENTAL TABLE** **S1** Details of the field experiments.

| **Trial details** | **Trial 1** | **Trial 2** | **Trial 3** |
| --- | --- | --- | --- |
| Type of plant stock | Clones | Clones | Clones |
| Location | Ibadan | Abuja | Abuja |
| Year | 2016 | 2016 | 2017 |
| Latitude | 07°29.639"N | 09°09.842"N | 09°09.842"N |
| Longitude | 003°54.092"E | 7°20.708"E | 7°20.708"E |
| Altitude (m) | 221m | 431m | 431m |
| Mean annual RF (mm) | 1411 | 1268 | 1268 |
| Average annual minimum Temp (^0^C) | 22.7 | 21.2 | 20.9 |
| Average annual maximum Temp (^0^C) | 31.8 | 31.8 | 31.5 |
| Field layout | Augmented row column | Augmented row column | Augmented row column |
| No. of blocks | 6 | 4 | 4 |
| No. of rows | 50 | 66 | 50 |
| No. of columns | 12 | 9 | 9 |

**SUPPLEMENTAL TABLE S2** Variance component and heritability estimates of six white Guinea yam traits by different genetic models.

|  |  | Traits^c^ | | | | | |
| --- | --- | --- | --- | --- | --- | --- | --- |
| Model^a^ | components^b^ | TTY | TTWPL | ATW | TTNPL | DM | YMV |
| Basic | Mean | 16.25±4.01 | 1.78±0.46 | 1.28±0.45 | 1.51±0.21 | 32.88±3.67 | 228.89±29.14 |
|  | ${}_{g}^{2}$ | 19.14±4.58 | 0.29±0.06 | 0.25±0.06 | 0.19±0.03 | 13.11±1.79 | 1282.55±112.78 |
|  | ${}_{b}^{2}$ | 5.17±1.66 | 0.05±0.02 | 0.04±0.02 | 0.006±0.005 | 3.47±0.82 | 68.55±34.31 |
|  | ${}_{e}^{2}$ | 17.97±4.36 | 0.21±0.05 | 0.15±0.06 | 0.17±0.03 | 9.39±1.57 | 331.58±96.03 |
|  | ${}_{p}^{2}$ | 42.28±2.29 | 0.55±0.03 | 0.45±0.03 | 0.36±0.01 | 25.97±1.59 | 1682.68±65.79 |
|  | H2 | 0.45±1.99 | 0.53±1.85 | 0.56±2.47 | 0.53±2.06 | 0.51±1.58 | 0.76±1.71 |
| A | ${}_{g}^{2}$ | 21.47±4.43 | 0.34±0.05 | 0.31±0.06 | 0.21±0.03 | 15.70±2.12 | 1334.40±106.76 |
|  | ${}_{a}^{2}$ | 3.88±2.49 | 0.06±0.04 | 0.10±0.05 | 0.06±0.02 | 5.22±2.52 | 137.57±68.90 |
|  | $\sigma_{\hat{g}}^{2}$ | 17.59±4.33 | 0.28±0.05 | 0.20±0.05 | 0.16±0.03 | 10.48±2.06 | 1196.83±106.27 |
|  | ${}_{b}^{2}$ | 4.17±1.56 | 0.03±0.02 | 0.03±0.02 | 0.005±0.03 | 3.24±0.77 | 46.47±22.91 |
|  | ${}_{e}^{2}$ | 17.15±4.02 | 0.19±0.05 | 0.13±0.05 | 0.16±0.03 | 8.77±1.50 | 319.56±80.46 |
|  | ${}_{p}^{2}$ | 42.79±2.45 | 0.55±0.03 | 0.47±0.03 | 0.37±0.02 | 27.91±1.63 | 1700.23±70.71 |
|  | h^2^ | 0.09±1.02 | 0.10±1.09 | 0.22±1.43 | 0.15±1.36 | 0.19±1.54 | 0.08±0.97 |
|  | H^2^ | 0.50±1.81 | 0.61±1.58 | 0.65±1.69 | 0.56±1.68 | 0.52±1.59 | 0.78±1.51 |
| A+D | ${}_{g}^{2}$ | 21.13±5.03 | 0.33±0.06 | 0.30±0.06 | 0.21±0.03 | 15.59±2.04 | 1389.46±111.04 |
|  | ${}_{a}^{2}$ | 0.175±0.82 | 0.007±0.006 | 0.09±0.05 | 0.05±0.03 | 4.51±2.41 | 67.39±44.38 |
|  | ${}_{d}^{2}$ | 20.93±5.03 | 0.16±0.09 | 0.06±0.07 | 0.06±0.03 | 1.29±1.24 | 572.35±216 |
|  | $\sigma_{\hat{g}}^{2}$ | 0.02±0.00 | 0.16±0.09 | 0.15±0.08 | 0.11±0.02 | 9.78±2.14 | 749.73±195.10 |
|  | ${}_{b}^{2}$ | 3.31±1.45 | 0.03±0.02 | 0.03±0.02 | 0.005±0.005 | 3.11±0.75 | 47.93±21.04 |
|  | ${}_{e}^{2}$ | 19.19±3.94 | 0.20±0.05 | 0.14±0.05 | 0.16±0.02 | 8.78±1.45 | 312.18±76.77 |
|  | ${}_{p}^{2}$ | 43.63±2.56 | 0.56±0.03 | 0.47±0.03 | 0.38±0.02 | 27.48±1.58 | 1749.579±80.92 |
|  | h^2^ | 0.004±0.38 | 0.01±0.18 | 0.19±1.56 | 0.12±1.51 | 0.16±1.52 | 0.04±0.55 |
|  | H^2^ | 0.48±1.96 | 0.60±1.67 | 0.64±1.78 | 0.57±1.57 | 0.57±1.29 | 0.79±1.34 |

^a^A, model fitted with additive variance–covariance structure; A+D, model fitted with additive plus dominance variance–covariance structure.

^b^ $\sigma_{g}^{2}$, total genetic variance; ${}_{a}^{2}$, additive genetic variance; ${}_{d}^{2}$, dominance variance; $\sigma_{\hat{g}}^{2}$, residual genetic variance; $\sigma_{b}^{2}$, environmental variance between plots within experiments; $\sigma_{e}^{2}$, within plot error variance; $\sigma_{p}^{2}$, individual phenotypic variance which is sum the variance components for the trait; h^2^, narrow-sense heritability; H^2^, broad-sense heritability.

^c^TTY, fresh tuber yield (t ha^-1^); TTWPL, fresh tuber yield per plant (kg); ATW, average tuber weight (kg tuber^-1^); TTNPL, tuber number per plant (count); DM, tuber dry matter content (%), YMV: yam mosaic virus severity score (AUDPC value).


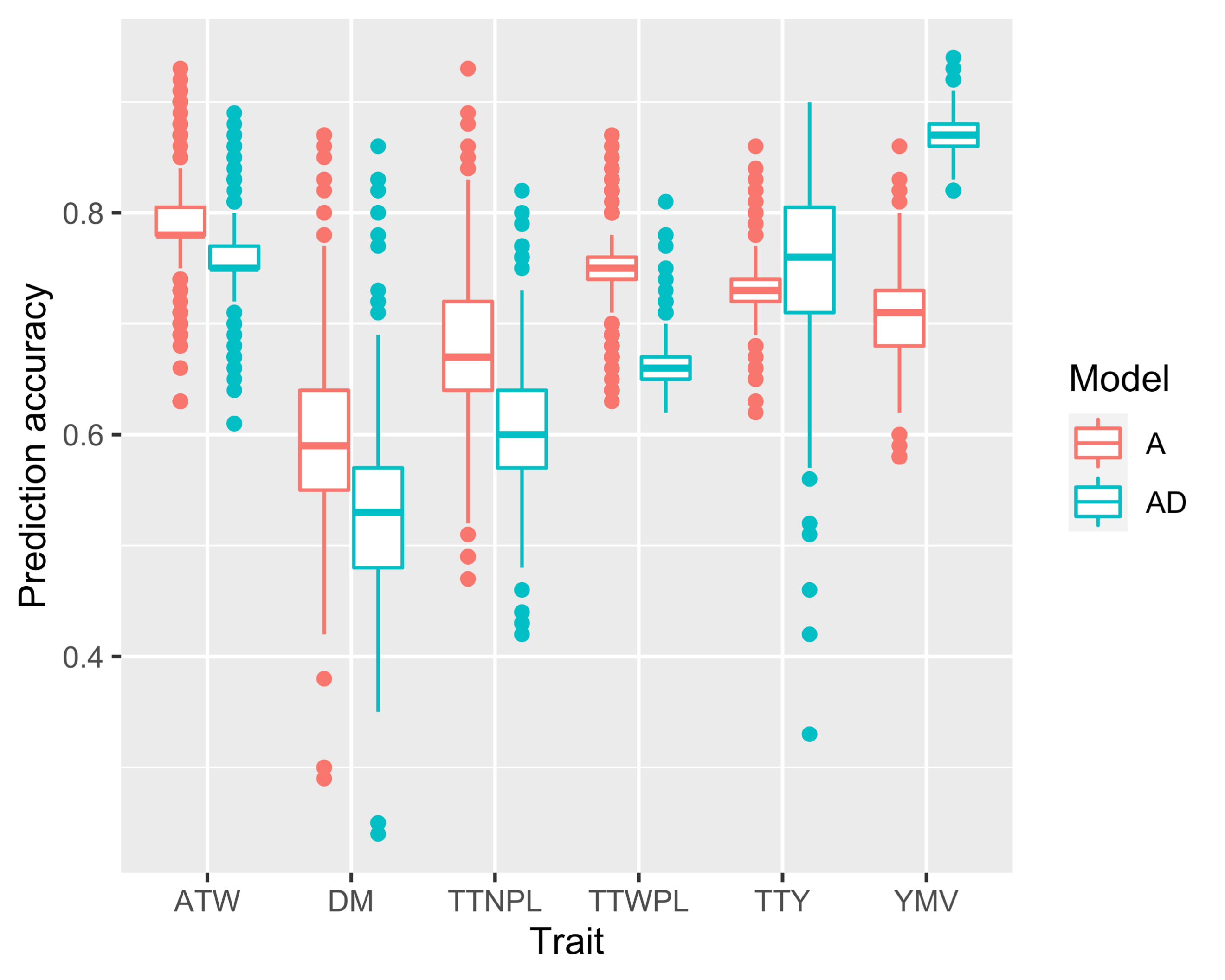


Supplementary figure S3. Prediction accuracy of two genetic models for each of the six yam traits
